# Non-classical functions of nuclear pore proteins in ciliopathy

**DOI:** 10.3389/fmolb.2023.1278976

**Published:** 2023-10-16

**Authors:** Yan Chen, Yuan Zhang, Xiangyu Zhou

**Affiliations:** ^1^ Obstetrics and Gynecology Hospital of Fudan University, Fudan University Shanghai Medical College, Shanghai, China; ^2^ Department of Assisted Reproduction, Shanghai First Maternity and Infant Hospital, Tongji University School of Medicine, Shanghai, China

**Keywords:** nuclear pore proteins, nucleoporins, Nup205, Nup188, cilia, ciliopathy, NEK3, left-right patterning

## Abstract

Nucleoporins (NUPs) constitute integral nuclear pore protein (NPC) elements. Although traditional NUP functions have been extensively researched, evidence of additional vital non-NPC roles, referred to herein as non-classical NUP functions, is also emerging. Several NUPs localise at the ciliary base. Indeed, *Nup188*, *Nup93* or *Nup205* knockdown results in cilia loss, impacting cardiac left–right patterning in models and cell lines. Genetic variants of *Nup205* and *Nup188* have been identified in patients with congenital heart disease and situs inversus totalis or heterotaxy, a prevalent human ciliopathy. These findings link non-classical NUP functions to human diseases. This mini-review summarises pivotal NUP interactions with NIMA-related kinases or nephronophthisis proteins that regulate ciliary function and explores other NUPs potentially implicated in cilia-related disorders. Overall, elucidating the non-classical roles of NUPs will enhance comprehension of ciliopathy aetiology.

## Introduction

The nuclear pore complex (NPC) resides within the nuclear envelope, merging the inner and outer membranes to create a channel. The overall NPC structure remains evolutionarily conserved with eight-fold rotational symmetry, comprising about 30 diverse nuclear pore proteins called nucleoporins (NUPs) (Fernandez-Martinez and Rout, 2021; Zimmerli et al., 2021). Comprising four ring scaffolds—the cytoplasmic ring (CR), inner ring (IR), nuclear ring (NR) and luminal ring (Lin et al., 2016; Beck and Hurt, 2017; Huang et al., 2022a)—the vertebrate NPC exhibits structural stability. The CR and NR share NUP components (Huang et al., 2022a), with the IR bridging them (Lin et al., 2016; Huang et al., 2022a; Huang et al., 2022b), whereas the luminal ring is situated in the ring lumen (Lin et al., 2016). Peripheral elements, cytoplasmic filaments and the nuclear basket connect to the CR and NR (von Appen and Beck, 2016).

The NPC’s nomenclature varies across species based on molecular mass. Most NUPs form robust subcomplexes within the NPC, including Y-complexes (also called the Nup84 complex in yeast and NUP107 complex in humans), NUP214 complexes (known as the Nup159 or Nup82 complex in yeast), NUP62 complexes and IR complexes (alo known as NUP93 complexes in humans) (Beck and Hurt, 2017). Linear motifs in NUPs connect major NPC modules (Lin et al., 2016; Beck and Hurt, 2017; Huang et al., 2022a). The structure studies from *Xenopus laevis* show that the NR comprises 10-member Y-complexes stabilised by Nup93 and Nup205 (Huang et al., 2022a). Each NR Y-complex has a short arm (Nup85, Nup43, and Seh1), long arm (Nup160, Nup37 and ELYS) and stem (Sec13, Nup96, Nup107, and Nup133) (Huang et al., 2022a). The CR contains extra Nup93 and Nup205 (inner Nup205) molecules, as well as five Nup358 molecules, but lacks ELYS (Beck and Hurt, 2017; Huang et al., 2022a). The IR comprises 30 molecules of nine distinct NUPs, including four Nup93, six Nup155, four channel NUP heterotrimers (Nup62/Nup58/Nup54) and two each of Nup205, Nup188, NDC1 and ALADIN (Huang et al., 2022b; von Appen and Beck, 2016; Stuwe et al., 2015).

In NPCs, NUPs can be divided into scaffold NUPs and phenylalanine–glycine (FG)-NUPs (Beck and Hurt, 2017). Scaffold NUPs form the NPC structure, anchoring the Nup62 complex and other FG-NUPs, and contribute to cytoplasmic filaments and the nuclear basket (Beck and Hurt, 2017; Fernandez-Martinez and Rout, 2021). FG-NUPs with phenylalanine–glycine-rich disordered domains interact with nucleocytoplasmic transport mechanisms (Beck and Hurt, 2017; Fernandez-Martinez and Rout, 2021), creating a size-selective diffusion barrier for molecules >40 kDa. They also bind nuclear transport receptors (Karyopherin and import/export proteins), facilitating signal-carrying cargo transport through the NPC (Beck and Hurt, 2017).

**FIGURE 1:**
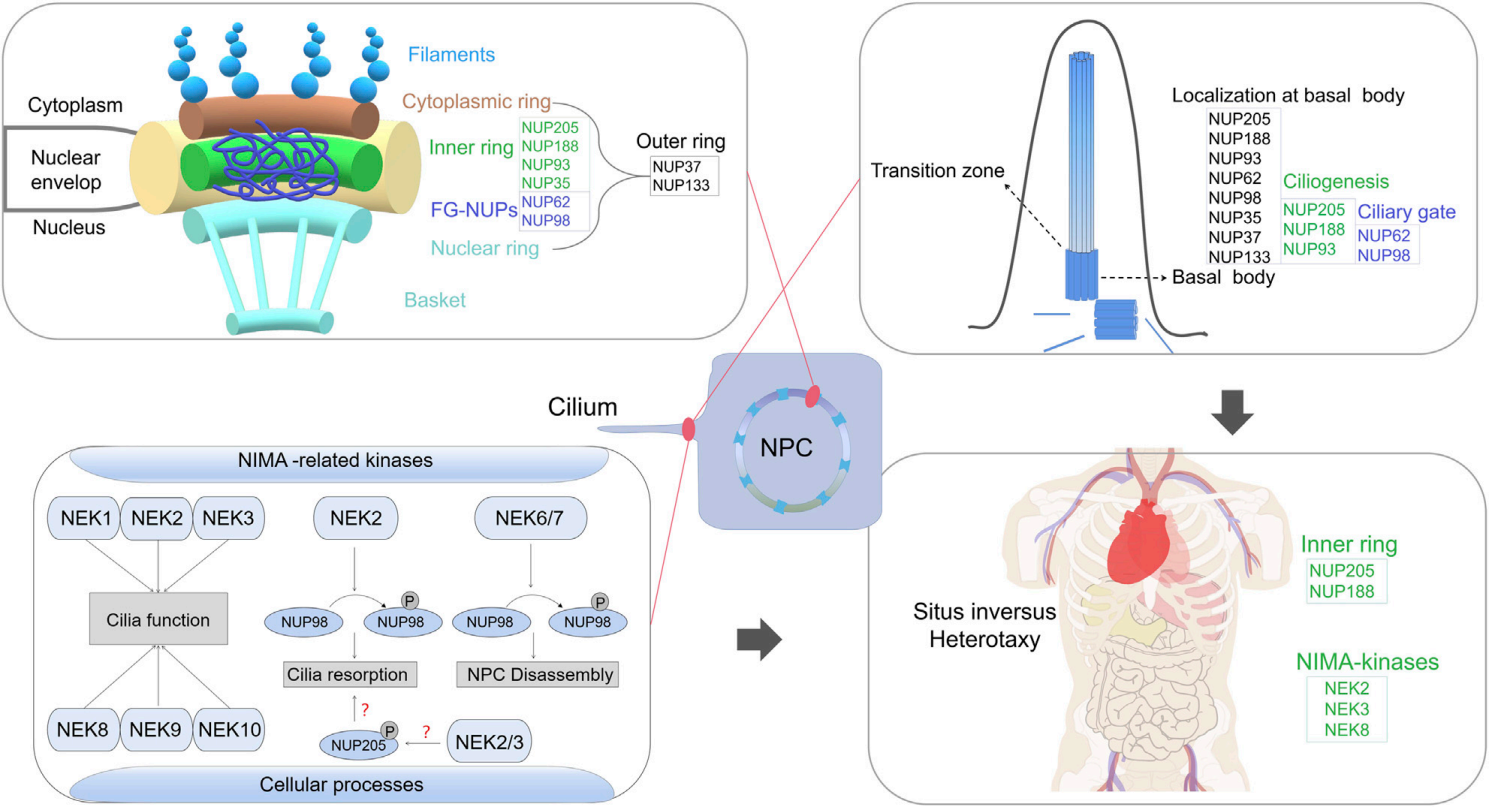
A comprehensive review of the emerging role of nucleoporins (NUPs) beyond their classical functions in nuclear pore complex assembly, with a particular focus on their involvement in ciliary processes and their interactions with NEK family in regulating cilium resorption, which link non-classical NUP functions to human ciliopathy.

Although NUPs are typically associated with NPC-related roles, they have been found in other subcellular components, including the kinetochore, centrosome, cilia base and chromatin (Kee et al., 2012; Mossaid and Fahrenkrog, 2015; Verhey and Yang, 2016). Notably, specific NUPs at cilia bases regulate transport between the cilia and cytoplasm (Kee et al., 2012; Takao et al., 2017). Moreover, mutations in some NUPs contribute to ciliopathies (Cardenas-Rodriguez and Badano, 2009; Del Viso et al., 2016). Certain NUPs, including NUP93, NUP188 and NUP205, play essential roles in cilia-related cardiac left–right (LR) patterning (Del Viso et al., 2016; Marquez et al., 2021). Inner and outer ring NUPs contribute differently to renal development, with dysfunction leading to nephrotic syndrome (Miyake et al., 2015; Braun et al., 2016) (Figure 1).

## Main text

### Roles of NUPs in cilia function

Cilia, conserved organelles extending from cell surfaces, crucially influence cell development and motor–sensory functions (Bisgrove and Yost, 2006; Berbari et al., 2009; Breslow et al., 2013). Distinct ciliary proteins regulate membrane–cytoplasmic transport at the ciliary gating zone (Breslow et al., 2013; Kee and Verhey, 2013; Diener et al., 2015). Cilia consist of a microtubule core, the axoneme, which extends from a modified centriole called the basal body. The axoneme is usually composed of microtubule doublets. However, each centriole is a cylinder of nine triplets of microtubules. (Bisgrove and Yost, 2006). The transition zone at the ciliary base, featuring a Y-shaped junction, serves as a gateway for proteins to enter and exit the ciliary compartment (Reiter et al., 2012; Diener et al., 2015). Cilia are categorised into motile and immotile/primary types based on movement and structure (Berbari et al., 2009). Regular motile cilia have a ring of nine peripheral microtubule doublets surrounding a central pair of single microtubules, i.e., a 9 + 2 microtubule arrangement, whereas primary or sensory cilia possess a 9 + 0 configuration and are immotile and present on most cell types (Kee and Verhey, 2013; Diener et al., 2015). Ciliary protein disruptions lead to ciliopathies, severe disorders affecting various organs (Bisgrove and Yost, 2006; Cardenas-Rodriguez and Badano, 2009). Ciliopathies result in diverse syndromes including situs abnormalities, respiratory infections, congenital heart disease (CHD), male infertility, nephronophthisis and neonatal cholestasis (Cardenas-Rodriguez and Badano, 2009; Chen et al., 2022a).

Interestingly, specific NUPs are suggested to localise to primary and motile cilia bases, with kinesin-2 motor KIF17 entry impeded by NPC transport inhibitors (Kee et al., 2012). Import to the ciliary compartment involves nuclear trafficking components, including importins, a Ran-guanosine triphosphate gradient and NUPs (Kee and Verhey, 2013). Transition zone proteins, including ciliopathy gene products, e.g., nephronophthisis (NPHP) and Meckel–Gruber syndrome (MKS) proteins, NUPs and septins, play gating roles (Takao et al., 2014; Yee et al., 2015). A ciliary pore complex (CPC) at the base, analogous to the NPC, has been hypothesised (Kee et al., 2012). Super-resolution imaging shows Nup188 clusters form two barrels at the cilium base (Del Viso et al., 2016). However, dimensions and organization of this barrel-like structure is incompatible with an NPC-like ring. Nanoscale NUP spatial organisation studies indicated that Nup93 and Nup188 lack the ∼100 nm diameter rings, suggesting these NUPs did not form the NPC-like rings at the cilium base. The clear difference between the organization of nups at the cilium base and that in the NPC argues against a proposed CPC model (Del Viso et al., 2016). Therefore, the CPC model remains controversial.

### NUP mutations and ciliopathies

Cilia dysfunction contributes to various ciliopathies, including hydrocephalus, polycystic kidney disease, retinal dystrophy and CHD (Bisgrove and Yost, 2006; Cardenas-Rodriguez and Badano, 2009; Marquez et al., 2021). In vertebrates, cilia-driven LR asymmetry in the heart is crucial during gastrulation, initiated by the ciliated LR organiser (LRO) (Fakhro et al., 2011; Blum et al., 2014). Both motile and immotile cilia break symmetry, ensuring organelle placement and vascular network development (Koefoed et al., 2014). Nodal signalling triggered by motile cilia’s leftward extracellular fluid flow and subsequent gene expression cascade establish LR asymmetry (Blum et al., 2014; Koefoed et al., 2014). The establishment of LR asymmetry defects leads to visceral malformations, termed heterotaxy (Htx) (Shiraishi and Ichikawa, 2012; Boskovski et al., 2013), often accompanied by severe CHDs (Shiraishi and Ichikawa, 2012). Cilia dysfunction is implicated in cystic kidney disease (Cardenas-Rodriguez and Badano, 2009; Chen et al., 2022a) and steroid-resistant nephrotic syndrome (SRNS), a common cause of chronic kidney disease, necessitating dialysis or transplantation due to progressive end-stage renal disease (Braun et al., 2016). Over 50 monogenic genes contribute to podocyte dysfunction in SRNS, indicating the involvement of multiple pathogenic signalling pathways (Braun et al., 2018).

Human genomics have advanced ciliopathy gene identification, and NUPs have gained prominence (Fakhro et al., 2011; Braun et al., 2018). IR nucleoporins, e.g., NUP93 and NUP188, participate in right LR patterning through cilium roles crucial for the heart’s LR asymmetry (Del Viso et al., 2016). Bi-allelic NUP205 mutations, likely NUP188 paralogs, are associated with CHD (Chen et al., 2019). Recessive NUP205 and NUP93 variants are linked to SRNS (Braun et al., 2016). Additionally, collaborations between NUPs and other proteins contribute to ciliopathies. Inherited polycystic kidney diseases (PKDs), including ADPKD, ARPKD, and NPHP, are linked to ciliopathies (Bisgrove and Yost, 2006). NPHP, recessive cystic kidney disease and MKS, characterised by renal–hepatic cysts and central nervous system malformations, involve transition zone localisation and interactions with certain NUPs (Bisgrove and Yost, 2006; Takao et al., 2017; Blasius et al., 2019). Nek2 expression in *Xenopus* LRO and kidneys links it to ciliopathies, with Nup98 interaction affecting cilium resorption (Fakhro et al., 2011; Endicott et al., 2015). Multiple NUPs found at cilia bases and mutations in NUPs are associated with nephrotic syndrome (Kee et al., 2012; Braun et al., 2018). Given the established connection of NUPs with cilia, investigating other NUPs’ roles in ciliopathies remains important.

### NUP205 and ciliary function

NUP205, a scaffold nucleoporin and NUP93 subcomplex member, resides in the IR of the NPC and is implicated in ciliary roles (Beck and Hurt, 2017; Huang et al., 2022b). Studies have highlighted NUP205’s potential significance in ciliary function (Chen et al., 2019; Marquez et al., 2021) (Table.1). Bi-allelic missense mutations (p.Thr1044Met and p. Pro1610Arg; NM_015135) in NUP205 were identified in a patient with situs inversus totalis (Chen et al., 2019). These mutations reduced protein interaction with NUP93 in patient-derived induced pluripotent stem cells (Chen et al., 2019). Nup205-silenced embryos showed disrupted cilia number and length in the retina, with around 30% displaying cardiac LR asymmetry defects (Chen et al., 2019). Subsequent research by Marquez et al. used morpholino oligos and CRISPR-based knockout in *Xenopus*, revealing abnormal cardiac LR patterning upon Nup205 depletion (Marquez et al., 2021). Morphants exhibited a 40% reduction in cilia number in the LRO, and two-cell stage embryos displayed cilia loss in multiciliate cells (MCCs) on the Nup205 MO-injected side compared with the control MO-injected side (Marquez et al., 2021). Additionally, GFP–NUP205 overexpression was localised in the cilium base in the *Xenopus* LRO (Marquez et al., 2021). Transmission electron microscopy confirmed mispositioned basal bodies in Nup205-depleted embryos (Marquez et al., 2021). Notably, NUP205 p. Pro1610Arg failed to rescue cilia loss, suggesting that it may be a loss-of-function allele (Marquez et al., 2021). Moreover, NUP205 p. Thr1044Met impacted protein stability, allowing overexpressed p. Thr1044Met to restore cilia levels (Chen et al., 2019; Marquez et al., 2021).

**TABLE 1:** The key components of the IR, including Nup205, Nup188, and Nup93, have been investigated in relation to cilia.

Nups	NPC	Localization in cilium	Genetic evidence from human diseases	Cilia-related phenotypes after knockdown
NUP205	IR	Base Marquez et al. (2021)	Bi-allelic missense mutations (Thr1044Met and Pro1610Arg) in situs inversus Chen et al. (2019)	Defects in LR asymmetry and heart-looping formation in zebrafish Chen et al. (2019); Reduced cilia length in human RPE cells Chen et al. (2019); Abnormal LR patterning and Dysfunctional pronephric development in *Xenopus* Marquez et al. (2021); Loss of cilia number in the LRO, epidermis, and pronephros in *Xenopus* Marquez et al. (2021)
			Homozygous missense mutation (Phe1995Ser) in SRNS Braun et al. (2016)	
NUP188	IR	Base Del Viso et al. (2016)	Rare copy number variations (gain of copy) in Htx Fakhro et al. (2011)	Abnormal LR cardiac morphologies in *Xenopus* Del Viso et al. (2016); Loss of cilia in mammalian cell lines and the LRO of *Xenopus* Del Viso et al. (2016)
			Bi-allelic LoF variants in patients with neurologic, ocular, and cardiac abnormalities Muir et al. (2020)	
NUP93	IR	Base Kee et al. (2012); Del Viso et al. (2016); Takao et al. (2017)	Homozygous missense mutations (Gly591Val and Tyr629Cys) in SRNS Braun et al. (2016)	Reductions in both cilia density and length in *Xenopus* Del Viso et al. (2016); The loss of cilia in mammalian cell lines and the LRO of *Xenopus* Del Viso et al. (2016); Significantly altered cardiac looping in *Xenopus* Del Viso et al. (2016)
NUP35	IR	Base Kee et al. (2012)	-	-

Beyond cardiac involvement, NUP205 influences kidney development. A homozygous missense mutation (p.Phe1995Ser) in NUP205 was linked to early-onset SRNS (Braun et al., 2016). Depleted Nup205 in embryos caused dysfunctional pronephric development and cilia loss (Marquez et al., 2021). Collectively supported by genetic evidence (biallelic or homozygous mutations of NUP205), cilia-related phenotypes (LR asymmetry defects and SRNS), the role of ciliogenesis (disrupted cilia number and length upon NUP205 depletion) and subcellular localisation at the cilium base (Braun et al., 2016; Chen et al., 2019; Marquez et al., 2021), previous findings underscore NUP205’s role in cilia bases and human ciliopathy. Moreover, NUP205’s potential interactions with two memers of NIMA (never in mitosis A)-related kinases, NEK2 and NEK3, vital for cilia-related abnormal cardiac LR patterning (Chen et al., 2019; Zhang et al., 2020), warrant further exploration. Notably, numerous NIMA paralogs are associated with ciliary function.

### NUP188 and ciliary function

The NUP93 subcomplex, comprising NUP93, NUP35, NUP188 and NUP205, forms a pivotal part of the inner ring of the NPC (Huang et al., 2022b). NUP188, likely a paralog of NUP205, establishes an exclusive direct interaction with NUP93. Initial insights into NUP188’s ciliary connection emerged from the study of Fakhro et al., who identified a NUP188 duplication in a ciliopathy patient with CHD and Htx (Fakhro et al., 2011). Depletion of Nup188 using morpholino oligos led to abnormal pitx2c expression, inducing cardiac looping defects akin to the Htx phenotype (Del Viso et al., 2016). Nup188 knockdown resulted in cilia loss in the LRO during embryonic development, although NPC function was largely preserved (Del Viso et al., 2016). Notably, overexpressing *Xenopus* or human NUP188 replicated a Htx-like phenotype in ∼15% of embryos, indicating that Nup188 overexpression mimics its loss of function (Del Viso et al., 2016). Endogenous Nup188 localisation at cilia bases reinforces their direct ciliary role. As mentioned earlier, contrary to the CPC hypothesis, super-resolution imaging exposed Nup188’s barrel-like structures at the cilium base, contrasting with NPC-like ring formation (Del Viso et al., 2016). Studies have positioned NUP188 below the transition zone as a constituent of pericentriolar material (PCM), directly interacting with CEP152, a PCM component (Del Viso et al., 2016; Vishnoi et al., 2020).

Prior research has highlighted the collaborative cilia-rescuing potential of Nup188 and Nup205, which may reciprocally restore cilia loss from Nup205 mutations or Nup188 morphants, implying their partially overlapped functions in MCC cilia (Marquez et al., 2021). Although NUP93 binds to either NUP205 or NUP188, they cannot be simultaneously bound, as shown *in vitro* reconstitution studies (Amlacher et al., 2011; Beck and Hurt, 2017). Nup188’s role in mitotic chromosome alignment has been documented (Itoh et al., 2013; Vishnoi et al., 2020). Importantly, patients harbouring recessive NUP205 and NUP188 mutations exhibited diverse clinical phenotypes. Bi-allelic truncating NUP188 variants manifested remarkably similar symptoms in six affected individuals, encompassing congenital cataracts, hypotonia, prenatal ventriculomegaly, white-matter anomalies, hypoplastic corpus callosum, CHDs and central hypoventilation (Muir et al., 2020). These individuals succumbed due to respiratory failure, with five not surviving their first year of life. Notably, CRISPR-mediated NUP188 knockout in *Drosophila* led to motor deficits and susceptibility to seizures (Muir et al., 2020), partially mirroring neurological symptoms observed in affected patients, suggesting that NUP188 and NUP205 may not be interchangeable in certain contexts.

### Role of NUP93 in cilia

As a pivotal member of the NUP93 subcomplex, NUP93 orchestrates the assembly of the CR/IR/NR through direct interactions with numerous NUPs. The presence of Nup93 at the cilium base has been reported (Kee et al., 2012). Engineered Nup93 versions, aimed at assessing interactions with transiting ciliary proteins, exhibited engagement at the cilium’s base and tip with cytosolic proteins, yet failed to interact with axoneme-associated motor KIF17 or transmembrane protein SSTR3. This localisation pattern indicates that Nup93 occupies a central niche in the inner–outer axis of the ciliary gating zone (Takao et al., 2017). Similar to NUP205 and NUP188, Nup93 depletion leads to cilia loss in the LRO, precipitating aberrant cardiac LR patterning in *Xenopus* (Del Viso et al., 2016). In nup93 morphants, pitx2c expression and classical LR signalling markers, such as COCO (also known as DAND5 or CERL2), exhibited notably abnormal patterns compared with control embryos (Del Viso et al., 2016). Pitx2, a homeodomain transcription factor, is important in regulating LR asymmetry of the internal organs. COCO is a Nodal antagonist involved in the establishment of the LR body asymmetry (Del Viso et al., 2016). Although these outcomes strongly support the functional involvement of NUP93 in cilia, no direct evidence yet substantiates the connection between NUP93 variations and human ciliopathies. Bi-allelic *NUP93* gene missense mutations have been identified in families with SRNS and congenital ataxia (Braun et al., 2016). These mutations decrease NUP205 levels in the NPC, disrupt NPC assembly and impair interaction with SMAD4 protein (a TGF-β signalling transcription factor) (Braun et al., 2016). However, the key cilia-related parameters, such as beating frequency and ultrastructure, in individuals bearing NUP93 mutations have not been investigated, despite nephrotic syndrome commonly being associated with ciliopathies.

### Roles of NUP62 and NUP98 in cilia

The NUP62 complex comprises NUP62, NUP54 and NUP58, categorized as FG-NUPs (Beck and Hurt, 2017). Positioned at the central channel of the NPC, FG-NUPs establish a selective nucleocytoplasmic barrier alongside scaffold NUPs (Beck and Hurt, 2017; Huang et al., 2022b). NUP62–EGFP constructs indicate the presence of NUP62 at the cilium, as confirmed by specific antibody detection at the ciliary base of epithelial cells (Kee et al., 2012). Within the ciliary gating zone, certain NUPs, as well as NPHP and MKS proteins, serve as essential components, orchestrating specialised gating mechanisms to regulate protein transit between the cilium and cytoplasm (Takao et al., 2014; Takao et al., 2017). The involvement of the channel nucleoporin Nup62 in facilitating ciliary entry of the cytosolic kinesin-2 motor KIF17, which directly engages with doublet microtubules of the axoneme, has been reported (Takao et al., 2017). This interaction leads to KIF17 relocating Nup62 to the cilium tip, spatially positioning Nup62 within the inner region of the ciliary gating zone (Takao et al., 2017). Notably, forced NUP62 dimerisation disrupts ciliary entry of most cytosolic proteins, whereas membrane protein gating remains unaffected (Takao et al., 2014). The dynamic nature of Nup62 within the ciliary gating zone, as established through FRAP assays, mirrors its behaviour in the NPC, indicating the structural and compositional adaptability of both nuclear and ciliary barriers (Sakiyama et al., 2016; Takao et al., 2017).

Nup98 is distinguished by its multiple FG sequences. Localization of Nup98 at the ciliary base has been reported, where it participates in regulating cilia length (Endicott and Brueckner, 2018). Employing a fluorescence-based diffusion trap system, it was demonstrated that Nup98 curbs the diffusion of soluble molecules exceeding 70 kDa into the cilium in cultured mammalian cells, signifying its role in restricting the influx of soluble macromolecules (Endicott and Brueckner, 2018). Although Nup98 knockdown does not disrupt the overall architecture of the NPC or the transition zone, it accelerates the diffusion rate of molecules exceeding 100 kDa into the cilium, consequently leading to reduced cilia length, which becomes more responsive to alterations in cytoplasmic soluble tubulin levels (Endicott and Brueckner, 2018).

Despite an accumulation of evidence highlighting the roles of NUP62 and NUP98 in cilia, there are as yet no direct indications from human diseases or model organisms to establish a clear link between NUP62/98 and ciliopathies. Depleting representative components of the central transport channel (Nup62) has no discernible effect on cardiac looping in *Xenopus* (Marquez et al., 2021). Although mutations in NUP62 and NUP98 have been identified in patients with autosomal recessive infantile bilateral striatal necrosis and Rothmund–Thomson-like spectrum (olombo et al., 2023; Basel-Vanagaite et al., 2006), evidence of a cilia-related connection to these diseases has not been reported.

### Potential roles of other NUPs in cilia

Apart from the aforementioned NUPs, NUP35 (an IR NUP), NUP37 and NUP133 (both outer ring NUPs) also exhibit localisation at the ciliary base (Kee et al., 2012). The presence of Nup85 is essential for the localisation of Nup98 at the ciliary base and for regulating cilia length (Endicott and Brueckner, 2018). Mutations in NUP107, NUP85, NUP133 and NUP160, encoding components of the outer ring subunits of the NPC, have been associated with SRNS, akin to Galloway–Mowat syndrome (Miyake et al., 2015; Rosti et al., 2017; Braun et al., 2018). However, despite nephrotic syndrome being a prevalent type of ciliopathy, the fundamental cilia-related parameters in affected patients remain unexamined.

### Interaction of NUPs with NEKs in cilia

Among primary cilia-related signalling pathways, NIMA-related kinases (Nek1–Nek11), a family of serine–threonine kinases, are implicated in diverse cellular processes (Fry et al., 2012). Although the precise roles of mammalian Nek proteins remain largely unclear, their frequent association with cilia is conspicuous. Nek1, Nek3 and Nek8 have been linked to primary cilium formation (Shalom et al., 2008; Manning et al., 2013; Chen et al., 2019). Rare genomic copy number variations in NEK2 have been identified in patients with Htx (Fakhro et al., 2011). Notably, Nek2 functions as a pivotal switch governing cilia biogenesis, crucial for normal LR patterning (Endicott et al., 2015). Loss of Nek8 in homozygous null mice results in randomised LR asymmetry (Otto et al., 2008). Remarkably, our previous research underscores the potential involvement of protein interactions between NUP205 and NEKs in disease onset and progression (Chen et al., 2019; Zhang et al., 2020). Other studies have revealed the significance of the Nek2–Nup98 interaction in regulating cilium resorption (Endicott et al., 2015). Mechanistically, the phosphorylation of NUP98 by various kinases, mainly NEK2/6/7, is crucial for NPC disassembly upon mitotic entry (Laurell et al., 2011) (Figure 1). Nup53’s phosphorylation diminishes its interaction with partner NUPs (Linder et al., 2017). Initiation of NPC disassembly can be mimicked by a blend of mitotic kinases, including NIMA, suggesting that phosphorylation-triggered nucleoporin dissociation is a key concept underpinning mitotic nuclear envelope permeabilisation (Linder et al., 2017). The activity of Nup205 is regulated through self-phosphorylation under normal physiological circumstances (Lu et al., 2014). Given the numerous potential phosphorylation sites on Nup205, its dynamics may be modulated by signalling- or cell cycle-dependent kinases, possibly including NEKs. Previously, we found that NUP205 (p.Thr1044Met) influences its own protein stability (Chen et al., 2019), prompting a deeper exploration into possible Nup205 phosphorylation and its implications for the regulation of cilia-related functions (Figure 1). Besides NEKs, NPHP proteins interact with NUPs at the base of primary cilia (Blasius et al., 2019). Disruption of NPHP genes impairs the anchoring of transition zone structures to the ciliary membrane and results in abnormal ciliary protein composition (Williams et al., 2011; Kee et al., 2012). Interaction analyses have revealed that Nup62 and the C-termini of NPHP4 and NPHP5 interact with the axoneme-associated kinesin-2 motor KIF17, while the N-termini of NPHP4 and NPHP5 interact with the transmembrane protein SSTR3 (Takao et al., 2017). Therefore, elucidating the potential effects of these interactions on protein activities that contribute to disease aetiology could be a promising strategy for achieving a deeper mechanistic understanding.

## Discussion

In this mini-review, we have summarised the latest research progress on the non-classical functions of NUPs that extend beyond their role in NPC assembly. In comparison to NUPs constituting other vital NPC components, such as the Y-complex, nuclear basket, cytoplasm and central channel, the key components of the IR, including Nup205, Nup188 and Nup93, have been extensively investigated in relation to cilia. Their subcellular localisation, contributions to ciliogenesis in model organisms and genetic evidence from population studies have provided insights into their involvement. Although direct evidence is lacking, certain components of the outer rings of the NPC, including NUP133 and NUP37, have been implicated in ciliary processes. However, not all NUPs localise to the ciliary base, e.g., NUP153 and NUP210. Thus, generating a high-resolution architecture of the cilium base in humans could effectively address debates surrounding the CPC model and provide insights into the specificity of NUPs in ciliary function.

Although ciliopathies can impact various organ systems, NUP-related diseases seem to preferentially affect cardiology and renal systems (Miyake et al., 2015; Braun et al., 2016; Braun et al., 2018; Chen et al., 2019; Burdine et al., 2020). Unlike many recessive loss-of-function mutations identified in classical cilia-related genes, e.g., DNAH and NKE family members, most pathogenic NUP mutations (excluding NUP188) in patients with SRNS or CHD are missense mutations (Miyake et al., 2015; Braun et al., 2016; Braun et al., 2018; Chen et al., 2019; Muir et al., 2020; Chen et al., 2022b). Nevertheless, five out of six affected individuals carrying NUP188 mutations died within their first year of life due to respiratory failure (Muir et al., 2020). This may be attributed to the crucial roles that most NUPs play in maintaining fundamental life activities, making them susceptible to loss-of-function mutations. We generated a Nup205 knockout mouse model and found that blocking Nup205 function resulted in severe developmental defects. Through external fertilisation, all homozygous Nup205 KO embryos arrested at the blastocyst stage, providing insight into why patient-identified NUP205 mutations are primarily missense (Braun et al., 2016; Chen et al., 2019). This observation is corroborated by loss intolerance probability (pLI) scores in the GnomAD database (Karczewski et al., 2020), where pLI signifies the probability of a gene belonging to the haploinsufficient class, with pLI >0.9 indicating extreme loss-of-function intolerance. NUP205 and NUP188 both exhibit haploinsufficiency with pLI scores of 1.00 and 0.95, respectively, whereas NUP93, NUP133 and NUP107 are fully tolerant to loss-of-function variants with pLI scores of 0. This finding indicates that NUP205 and NUP188 possess more pivotal biological functions from an evolutionary perspective compared with other NUPs.

Ultimately, the identification and characterisation of NUPs at the ciliary base will provide novel insights into the precise mechanisms underlying cardiac and renal pathologies. Establishing the causal relationship between NUP variants and cilia-related disorders represents a crucial step toward developing advanced therapeutic strategies that enhance patient longevity and quality of life. In summary, this mini-review reinforces not only the role of NUPs in the context of cilia but also the importance of these functions in human diseases.
